# Metabolomics for hematologic malignancies: Advances and perspective

**DOI:** 10.1097/MD.0000000000039782

**Published:** 2024-09-20

**Authors:** Xinglan Li, Mengyu Xu, Yanying Chen, Yongqing Zhai, Junhong Li, Ning Zhang, Jiawei Yin, Lijuan Wang

**Affiliations:** a Linyi People’s Hospital, Shandong Second Medical University, Linyi, PR China; b Hematology Laboratory, Linyi People’s Hospital, Linyi, PR China; c Department of Orthopedics, Linyi People’s Hospital, Linyi, PR China; d Department of Anesthesiology, Linyi People’s Hospital, Linyi, PR China; e Central Laboratory, Linyi People’s Hospital, Linyi, PR China; f Key Laboratory of Tumor Biology, Linyi, PR China; g Key Laboratory for Translational Oncology, Xuzhou Medical University, Xuzhou, PR China; h Department of Hematology, Linyi People’s Hospital, Linyi, PR China.

**Keywords:** amino acid metabolism, glucose metabolism, leukemia, lipid metabolism, lymphoma, myeloma

## Abstract

With the use of advanced technology, metabolomics allows for a thorough examination of metabolites and other small molecules found in biological specimens, blood, and tissues. In recent years, metabolomics has been recognized that is closely related to the development of malignancies in the hematological system. Alterations in metabolomic pathways and networks are important in the pathogenesis of hematologic malignancies and can also provide a theoretical basis for early diagnosis, efficacy evaluation, accurate staging, and individualized targeted therapy. In this review, we summarize the progress of metabolomics, including glucose metabolism, amino acid metabolism, and lipid metabolism in lymphoma, myeloma, and leukemia through specific mechanisms and pathways. The research of metabolomics gives a new insight and provides therapeutic targets for the treatment of patients with hematologic malignancies.

## 1. Introduction

The metabolomics is a more sensitive and accurate tool that can capture the micro changes of gene and protein expression and amplify them in the form of metabolites, which is different from genomics and proteomics. Various methodologies are employed by the metabolomics field, including the utilization of separation techniques, cutting-edge instrumentation, and sophisticated data processing methods. The challenge of metabolomics analysis is the rapid temporal dynamic changes in analyte and sample composition, reflecting changes in endogenous and exogenous species such as drugs, toxins, microorganisms, and nutrients.^[1]^ Yet, the ability of proteomic analysis to differentiate biological organisms by their protein sequences is frequently highlighted^[2]^ (Especially beneficial in microbiome investigations). Nevertheless, the identification of species in metabolomics encounters difficulties due to the shared occurrence of small molecules in diverse organisms.^[3]^ However, this can facilitate the sharing of knowledge about the physical characteristics that guide identification among species, thus contributing to the study of metabolomics animal modeling. Another major difference between proteomics and metabolomics techniques relates to the interpretation of fragmentation data. By utilizing known protein sequences and enzymatic degradation patterns, researchers can anticipate peptide sequences and predict fragmentation profiles. Furthermore, due to the complex structure of proteins, it is common to observe the presence of multiple peptides, providing a higher level of confidence in identifying the proteins. In a different scenario, metabolic studies present a unique challenge due to the varying sizes and complex molecular structures of metabolites, resulting in inconsistent fragmentation patterns for individual species. Up to now, it has been widely observed that notable alterations in metabolism emerge throughout the process of tumor re-metastasis, providing continuous metabolic support to tumor cells. It has been demonstrated that these alterations in metabolism play a critical function in the progression of tumors, aiding cells in adjusting to variations in the pharmacological setting, potentially fostering cellular resistance to drugs.^[4–6]^ The formation of metabolites is a reflection of the interaction between biological and environmental elements, presenting significant potential in connecting genotypic and phenotypic information. Therefore, this has led to the gradual adoption of metabolite analysis as an increasingly sensitive indicator of an individual’s biological condition. The hematologic malignancies mainly include lymphoma, myeloma and leukemia, with complex clinical classification and strong heterogeneity.^[7]^ The key to the treatment of hematologic malignancies is early diagnosis and personalized treatment based on the type of tumor. Metabolic abnormalities also exist in hematologic malignancies, and the accuracy of metabolomics provides a possibility for early diagnosis. This article delves into the involvement of metabolomic pathways and networks related to glucose metabolism, amino acid metabolism, and lipid metabolism in various hematologic malignancies.

## 2. Metabolomics

### 2.1. Glucose metabolism

According to the Pasteur effect, glycolysis enters the carboxylic acid cycle, producing adenosine triphosphate (ATP) under aerobic conditions and lactic acid under anaerobic conditions. In the context of glycolysis, lactate is generated not only as a result of this process but also as a means to fuel the tricarboxylic acid (TCA) cycle for tumor cell regulation and induce histone lactylation, transitioning anti-inflammatory type 1 TAMs to pro-inflammatory type 2 TAMs. Additionally, there is another branch of glycolysis known as the pentose phosphate pathway (PPP) that yields pentose. The *R*-5-P generated by the PPP increases the rate at which nucleic acids are synthesized in cancer cells.^[8]^ This heightened activation of the PPP in cancer cells serves to mitigate the harmful impact of active reactive oxygen species (ROS) and contributes to the provision of essential materials for DNA replication.^[9]^ The rapid consumption of glucose by cancer cells leads to the creation of a glucose-deficient and lactic acid-abundant environment, compromising the effectiveness of CD8 + T cells and NK cells in fighting tumors,^[10]^ thus promoting tumorigenesis.

### 2.2. Amino acid metabolism

Aside from serving as supplies of carbon and nitrogen for the production of biomolecules like nucleotides, and their metabolites can join the TCA cycle and supply cells with ATP. Tumor cells can compete for nutrients to consume amino acids in the microenvironment, resulting in the suppression of T cell immunity, meanwhile, the detrimental byproducts of amino acid metabolism can also hinder the function of t cells. What’s more, the conversion process of amino acid metabolism leads to the production of pyruvate, partially facilitating the process of glycolysis and leading to the production of lactic acid, this process rapidly generates energy^[11]^ regulates glucose and glutamine metabolism to produce α-KG regulates lipid metabolism to interfere with T cell,^[12]^ leading to tumor immune escape.

### 2.3. Lipid metabolism

Lipid metabolism imbalance is a significant metabolic changes in cancer. Throughout the progression of tumors, there is a constant fluctuation in nutritional availability. This process is utilized by cancer cells to acquire the necessary energy, biofilm components, and signaling molecules for their various functions including proliferation, survival, invasion, and so on.^[13]^

## 3. Metabolomics and lymphoma

Lymphoma is arising from lymph nodes and lymphoid tissues, which is divided into two main types: Hodgkin’s lymphoma (HL) and non-Hodgkin’s lymphoma (NHL). NHL is the main type of lymphoma and mainly includes diffuse large B-cell lymphoma, follicular lymphoma and Burkitt’s lymphoma.^[14,15]^ Currently, chemotherapy and immunotherapy are the main treatments for lymphoma patients, but most patients cannot be cured. A research analyzed the metabolomics of two blood cancers using plasma samples from patients with myeloma and NHL, as well as healthy individuals. The analysis aimed to identify all potential metabolites and pathways affected by LC-MS metabolomics. The findings indicated notable variances in metabolomic profiles when comparing individuals with myeloma and NHL to those in the healthy control group. These differences implicated disruptions in various metabolic routes, including choline metabolism and oxidative phosphorylation, which were correlated with the advancement and proliferation of tumors.^[16]^ Some researchers gathered urine specimens from individuals diagnosed with NHL, utilizing non-targeted GC-MS for metabolic assessments, a recognized method for distinguishing between various test groups. The results showed that various metabolites helped to differentiate between healthy subjects and patients with diffuse large B-cell lymphoma (DLBCL).^[17]^ An emerging study in metabolomics offers a pathway to unearth fresh indicators for identifying and characterizing a range of lymphoma subtypes. Analysis of plasma samples obtained from patients diagnosed with diverse forms of lymphoma was conducted using gas chromatography-mass spectrometry. Elevated levels of elioic acid and hypoxanthine (HX) were observed in individuals diagnosed with HL, Multiple myeloma (MM), chronic lymphocytic leukemia (CLL), and DLBCL compared to control subjects across all research cohorts.^[18]^ Yoo et al^[19]^ analyzed urine samples collected from individuals diagnosed with various types of lymphomas, and then processed the information into low-mass ions. Three ion peaks with low mass and high intensity were chosen for examination, one of which was found at 137.08 m/z ion range detected as HX. Intracellular levels of HX and xanthine exhibited an inverse relationship with the modulation of adenylate energy, consequently affecting cellular ATP levels. Given the identification of abnormal metabolic processes as early signs of lymphoma, further investigations are warranted. Utilizing metabolomics allows for the exploration of novel indicators and the advancement of potential noninvasive diagnostic and prognostic instruments.^[20]^ The understanding of lymphoma pathogenesis can be significantly enhanced through the utilization of metabolomics, leading to the creation of more sensitive diagnostic methods and effective therapeutic interventions.

### 3.1. Metabolomics in diffuse large B-cell lymphoma

Accounting for 30% to 40% of all NHL cases, DLBCL exhibits a highly aggressive nature. The initial treatment commonly used in clinics for DLBCL is the R-CHOP regimen. Nevertheless, a significant proportion of patients, approximately 40% to 50%, encounter relapse post-treatment and progress to resistant DLBCL. Due to the considerable diversity and reoccurrence percentage of DLBCL, the exploration of highly precise and responsive biomarkers as fresh therapeutic targets may offer novel avenues for the diagnosis, management, and prediction of DLBCL. Research findings are increasingly indicating that metabolomics plays a significant role in influencing the onset and advancement of DLBCL, allowing for dynamic analysis of disease progression and serving as a valuable biomarker for the identification of DLBCL.

#### 3.1.1. Glucose metabolism

**Figure 1.**
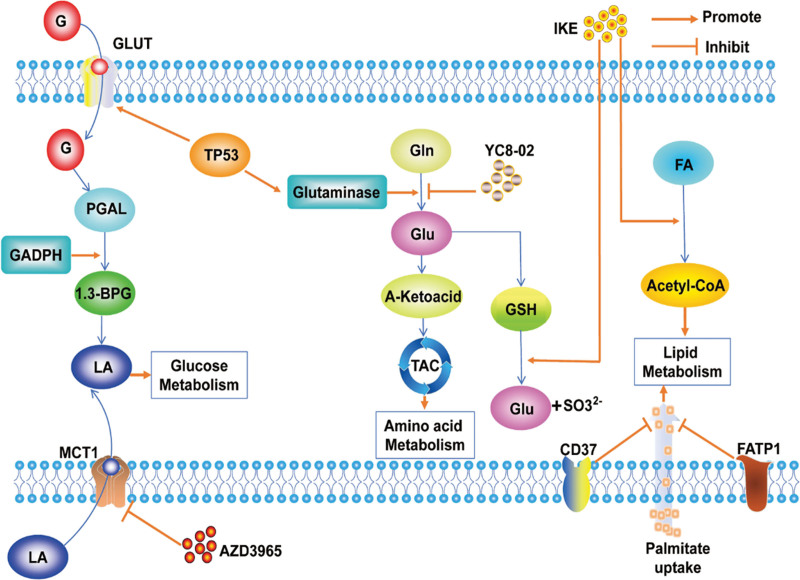
Glucose, amino acid and lipid metabolism in DLBCL. Glucose enters the cell via GLUT and produces lactate through glycolysis, the TP53 activates the GLUT, thereby promoting the development of DLBCL. AZD3965 Inhibiting MCT 1 for LA and H + exchange to inhibit the occurrence of DLBCL. TP53 activates glutaminase to promoting glutamine degradation, enters TCA, and promoting the development of DLBCL, While YC 8-02 showed the opposite. IKE promotes lipolysis, as well as the GSH transformation. 1, 3-BPG = 1, 3-diphosphoglycerate, DLBCL = diffuse large B-cell lymphoma, FA = fatty acid, G = glucose, Gln = glutamine, Glu = glutamic acid, GLUT1 = glucose transporter-1, GSH = glutathione, LA = lactic acid, PGAL = 3-phosphoglyceraldehyde, TAC = tricarboxylic acid cycle, TCA = tricarboxylic acid.

A critical component of the glycolytic process is Glyceraldehyde-3-phosphate dehydrogenase, which can promote the progression and metastasis of DLBCL by facilitating glycolysis as well as increasing the level of glycolysis to provide sufficient energy. A distinctive property of glyceraldehyde-3-phosphate dehydrogenase is its capability to assess DLBCL patients undergoing R-CHOP treatment and serve as a reliable independent indicator for predicting enhanced prognosis among patients^[21]^ (Fig. 1). Research has shown that Myc, a prominent regulator of metabolic processes, induces genes that coordinate glucose and amino acid catabolism and control nutrient and metabolite transport in Eμ-Myc mice, contributing to the advancement of DLBCL.^[22]^ Functional mutations in TP53 can affect glucose metabolism, which promotes DLBCL progression by activating glucose transporter protein (GLUT), glutaminase and fructose 2,6-bisphosphatase, inducing a metabolic shift to aerobic glycolysis and free radical generation^[23]^ (Fig. 1). Thus, increased expression of glycolysis-related enzymes and Myc, as well as mutations in TP53, can generate more energy by increasing the level of glucose metabolism and are key factors affecting disease progression and poor prognosis in DLBCL.

Progress has also been made in the clinical treatment of DLBCL with glucose metabolism. DLBCL shows high expression of the monocarboxylate transporter 1 (MCT1), which acts as a crucial controller of glucose metabolism, thereby stimulating the proliferation of DLBCL tumors both in laboratory settings and within living organisms. AZD3965, an MCT1 inhibitor, inhibits MCT1 expression and then affects glucose metabolism by reducing glycolytic intermediates production and blocks the tricarboxylic acid cycle by inhibiting the pyruvate dehydrogenase activity^[24]^ (Fig. 1). A latest study suggests that AZD3965 combined with the mitochondrial respiratory complex I inhibitor IACS-010759 can synergistically induce DLBCL cell death in vitro.^[25]^ Research on inhibitors related to glycolysis pathways provide new ideas for the treatment of DLBCL.

#### 3.1.2. Amino acid metabolism

Amino acid metabolism provides adequate nutrition and energy for DLBCL cell survival, growth, and metastasis. Supporting healthy and malignant cell growth, the crucial metabolic pathway of serine synthesis plays a vital role. A disruption in the first key enzyme, glycerol phosphate dehydrogenase (PHGDH), within this pathway can result in abnormalities in hair growth and compromised production of antibodies with heightened affinity. Moreover, increased activity of enzymes participating in the serine biosynthetic process characterizes lymphoma derived from germinal center B-cells, with elevated PHGDH levels correlating with unfavorable outcomes in diffuse large B-cell lymphoma.^[26,27]^ Furthermore, the regulation of various metabolic pathways, such as glycolysis and glutaminolysis, by MYC includes a significant emphasis on the serine synthesis pathway as a crucial hub for cellular proliferation. This pathway utilizes 3-phosphoglycerate, a product downstream of glycolysis, in the production of serine through the collaborative action of PHGDH, phosphoserine aminotransferase 1, and phosphoserine phosphatase.^[28]^ This observation points towards the essential role of PHGDH in the immune response, particularly in the context of lymphoma treatment.^[26]^ There is also a new evidence that glucose and aspartate are closely associated with the development and pathogenesis of DLBCL, and plasma methionine and cysteine are significantly decreased in DLBCL patients with poor prognosis, and branched-chain amino acids are continuously decreased.^[29]^ Meanwhile Stenson et al reported that valine was a candidate biomarker for DLBCL prognosis.^[30]^

Studies in the clinic have demonstrated the pivotal role of abnormal glutamine metabolism in the development of drug resistance in DLBCL. sirtuin 3 induces enhanced glutamine utilization, promoting B-cell lymphangiogenesis in DLBCL through a non-oncogene mechanism triggered by metabolism. Interruption of glutamine utilization by targeting the mitochondrial class I sirtuin inhibitor YC8-02 induces DLBCL cell death.^[31]^ The discovery of this inhibitor provides a new avenue for the treatment of DLBCL. These findings indicate that the exploration of amino acid metabolism plays a crucial role in uncovering the pathogenesis of DLBCL and offers valuable insights for therapeutic approaches (Fig. 1).

#### 3.1.3. Lipid metabolism

The metabolic reprogramming in cancer cells goes beyond reliance on glycolytic conversion, the most dynamic metabolic process in numerous cancer forms involves the metabolism of fatty acids (FA). Recent findings indicate that there is an interaction between CD37 and FA transporter protein 1, leading to the inhibition of exogenous palmitate uptake. Knock-out of CD37 enhanced the lipid storage capacity in DLBCL cells, which lead to an increased dependence of cells on FA-metabolic switch.^[32]^ Targeted disruption of CD37-FA transporter protein 1 interaction using metabolic inhibitors may decrease survival and proliferation of DLBCL (Fig. 1).

A study using untargeted mass spectrometry (MS) in conjunction with ultra performance liquid chromatography (UPLC) analyzed lipid changes in DLBCL cell response to imidazolone-erastin (IKE) treatment, the results demonstrating that IKE, an erastin analog with high water solubility and potency, acts as a ferroptosis inducer, accelerates the consumption of glutathione (GSH) and lipid peroxidation through inhibition of system xc−, reduces the cell toxicity and exerts antitumor effects^[33]^ (Fig. 1). Clinical metabolomic analysis of patient plasma and cell lines demonstrated that levels of choline intracellular metabolites were upregulated in lysine deacetylase inhibitors panobinostat treated cells. Further investigation into the mechanism unveiled that panobinostat stimulates the choline pathway by triggering the PI3K pathway via choline kinase alpha. playing a significant role in the choline metabolic process.^[23,34]^ These discoveries suggest a potential novel approach in treating DLBCL through the choline pathway post KDACI therapy.

### 3.2. Metabolomics in Burkitt’s lymphoma

Burkitt’s lymphoma (BL) is a rare but highly aggressive type of non-Hodgkin lymphoma, primarily derived from b lymphocytes, characterized by rapid growth and systemic metastasis.^[35]^ The mortality rate of BL patients is high, and the treatment effect of surgery and chemotherapy is poor. Advancing the exploration of BL pathogenesis through metabolomics studies can offer a solid foundation for therapeutic decision-making.

#### 3.2.1. Glucose metabolism

**Figure 2.**
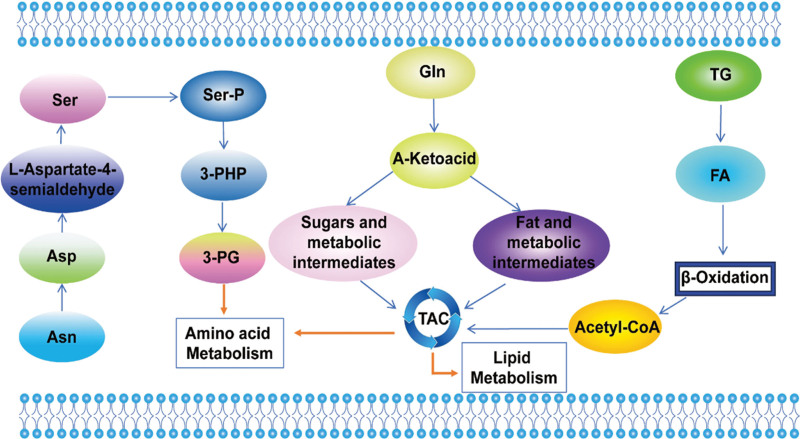
Glucose, amino acid and lipid metabolism in BL. Glycolysis may rapidly deplete blood glucose to promote proliferation and growth of tumor cells. Increased triglyceride metabolism leads to elevated levels of glycerol and FA, which can be converted to acetyl-CoA for the TCA cycle through β-oxidation, finally provides energy for BL. 3-PG = 3-phosphoglycerate, acetyl-CoA = acetyl coenzyme A, Asn = asparaginate, BL = Burkitt's lymphoma, FA = fatty acid, Gln = glutamine, Ser = serine, Ser-P = phosphoserine, TCA = tricarboxylic acid, TG = triglyceride.

One of the characteristics of BL is the abnormal activation of MYC gene caused by t(8; 14)(q24.1; q32) rearrangement. With the help of U-(13)C-glucose stable isotope-resolved metabolomics, Anne Le et al^[36]^ observed that an elevated expression level of MYC increased the glucose consumption and lactate production in human BL P493 cell line, the levels of fumarate, malate, and citrate derived from glutamine were significantly increased in glucose deficiency. The U-(13)C-labeling patterns demonstrated a new mechanism for the glucose-independent TCA cycles in BL. Through the utilization of comprehensive gene expression profiling analysis, the study conducted by Irene Bagaloni et al^[37]^ unveiledan upregulation of genes related to cellular glucose metabolism in BL cells, leading to an increase in the aerobic glycolysis pathway and a decrease in the TCA cycle(Fig. 2), furthermore, research revealed that metformin has the ability to trigger apoptosis in BL cells by upregulating GLUT1, pyruvate kinase M1/2, and lactate dehydrogenase A, which are closely linked to cellular glucose metabolism. There is a study that the serum metabolomics of BL mouse model based on nuclear magnetic resonance technology, and the results showed that the blood glucose level of tumor bearing mice was lower and the serum lactate level was higher, suggesting that glycolysis can rapidly consume blood glucose and promote the proliferation and growth of tumor cells.^[38]^ This phenomenon is consistent with the “Warburg effect.” These potential biomarkers identified in the above-mentioned studies could offer valuable insights into the diagnosis and prognosis of BL.

#### 3.2.2. Amino acid metabolism

Using a tracer-based approach and differentially expressed genes from RNA sequencing datasets, Eraslan et al^[27]^ confirmed that the genes involved in serine synthesis were higher in BL than in DLBCL, indicating that BL consumes more extracellular asparagine, the extracellular asparagine not only regulates serine uptake, but also increases serine production, inhibited the serine biosynthesis pathway combined with anticancer drug asparaginase may be more effective treatment for BL.

#### 3.2.3. Lipid metabolism

The increased metabolism and oxidation capacity of FA can provide the necessary biological energy for the rapid proliferation and expansion of tumor cells. Increased triglyceride metabolism leads to higher levels of glycerol and FA, which can be transformed into acetyl coenzyme A for the TCA cycle through β-oxidation, finally provides energy for BL^[32,38]^ (Fig. 2).

### 3.3. Metabolomics in follicular lymphoma

Follicular lymphoma (FL) is a malignancy arises from germinal center B cells that accounts for nearly 20% of all lymphomas, and typically manifests as painless lymph node enlargement, capable of infiltrating any part of the body or transforming into more aggressive forms like diffuse large B-cell.^[39]^ Recent years have seen the application of metabolomics in identifying altered characteristics indicative of pathophysiologic conditions, thereby offering insights into pharmacological interventions or external influences, ultimately contributing to the advancement of FL treatment.

#### 3.3.1. Glucose metabolism

**Figure 3.**
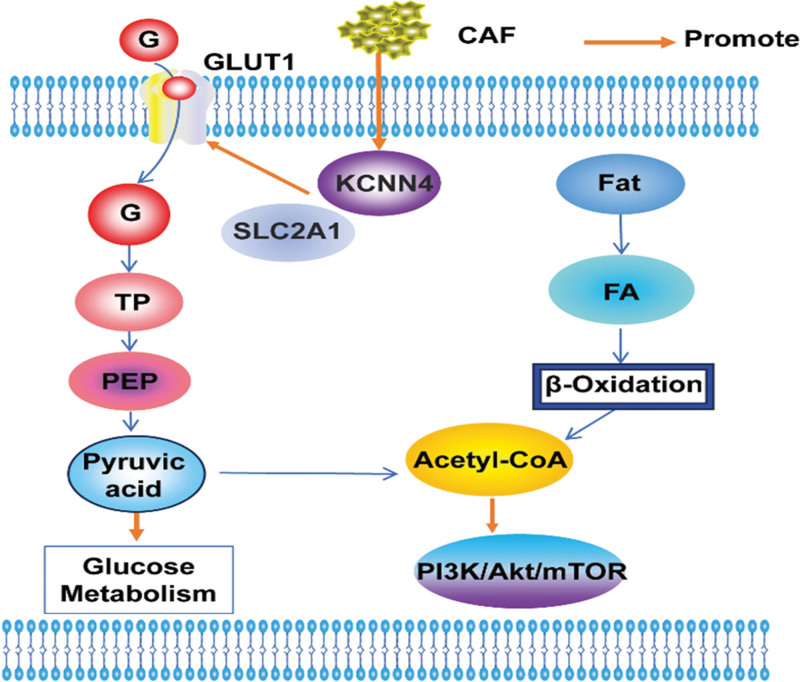
Glucose and amino acid metabolism in FL. KCNN4, SLC2A1 Activation of GLUT 1 promotes glycolysis, while CAF promotes this process, generates acetyl-CoA, activates the PI3K/Akt/mTOR pathway, and promotes the occurrence of FL. Lipid metabolism generates acetyl-CoA, which enters the above pathway. CAF = cancer-associated fibroblasts, FL = follicular lymphoma, KCNN4 = potassium calcium-activated channel subfamily N member 4, PEP = phosphoenolpyruvate, SLC2A1 = solute carrier family 2 member 1, TP = total phosphorus.

The ion channels and transport proteins provide a new insight for the glucose metabolism study in FL. With the help of cDNA microarray data, Alberto Magi et al^[40]^ found that ion channels and transport proteins related genes potassium calcium-activated channel subfamily N member 4 (KCNN4) and solute carrier family 2 member 1 (SLC2A1) were high expressed in FL and SLC2A1 is a key gene that connect channels and transporters in FL. While in relapsed FL patients, SLC2A1 and KCNN4 were decreased, which suggested a down-regulation of glycolysis progress. The metabolic changes may contribute to relapsed FL treatment. Glucose metabolism play an important role in FL tumor microenvironment (TME). Yuwei Deng group^[41]^ showed that GLUT1 was highly expressed in FL patients who progressed within 24 months, promoting glycolysis within the TME, increasing the infiltration of various suppressive immune cells, and ultimately activating the PI3K/Akt/mTOR pathway, accelerating FL progression (Fig. 3).

#### 3.3.2. Amino acid metabolism

Amino acid metabolism also play an important role in FL TME. As the key component of the TME, cancer-associated fibroblasts can isolate from FL samples. Functional study found that cancer-associated fibroblasts can increase anaerobic glycolysis and secrete pyruvate, reduce intracellular reactive oxygen species production, thereby promoting tumor cell survival. In addition, mechanism analysis found that inhibition of pyruvate uptake/outflow by the monocarboxylate transporter (MCT) inhibitor α-cyano-4-hydroxycinnamic acid increases intracellular ROS production and decreases lymphoma cell survival^[42]^ (Fig. 3).

## 4. Metabolomics and myeloma

MM is a plasma cell tumor that accounts for 15% of hematologic cancers.^[43]^ It is characterized by the aggregation of abnormal plasma cells in the bone marrow (BM) environment and the presence of monoclonal immunoglobulins in the blood or urine, causing bone destruction, anemia, renal impairment, and abnormal immune function. Current treatments for myeloma include multiple drug combinations, such as the proteasome inhibitor bortezomib, the immunomodulatory agents lenalidomide and the steroid dexamethasone, monoclonal antibodies targeting tumor cells, and autologous stem cell transplantation, which have greatly improved patient survival, but most patients inevitably experience disease progression or multiple relapses during the course of the disease.^[44]^

### 4.1. Glucose metabolism

**Figure 4.**
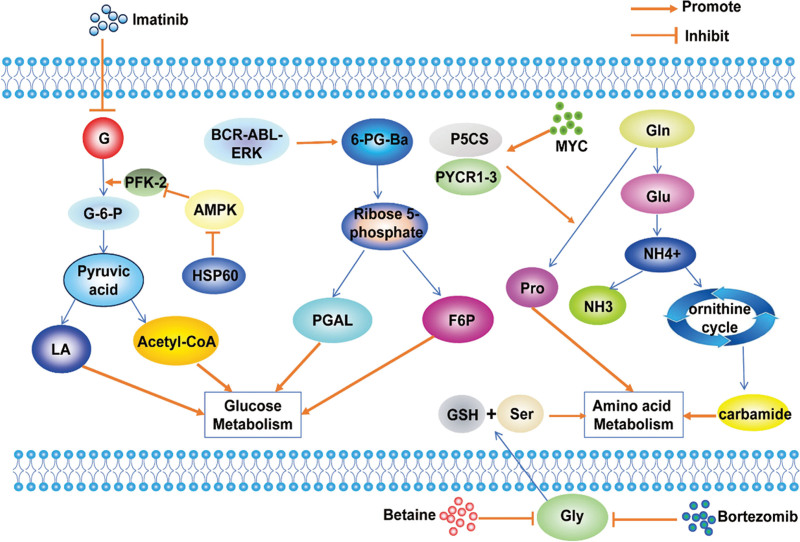
Glucose, amino acid and lipid metabolism in MM. BCR-ABL-ERK signaling drives the PPP and RNA biosynthesis in myeloma cells and that imatinib inhibits glycolysis and PPP uptake of glucose via acetyl-CoA and NADPH resulting in PPP damage and RNA nucleotide accumulation and negative regulation of mRNA, and HSP60 inhibits AMPK, which in turn inhibits PFK-2 and inhibits glycolysis. MYC increases the expression of PYCR1-3 and P5CS, thereby stimulating glutamine to proline biosynthesis. F6P = fructose-6-phosphate, G-6-P = Glucose-6-phosphate, Gly = glycine, GSH = glutathione, LA = lactic acid, MM = multiple myeloma, P5CS = pyrroline-5-carboxylate synthase, PGAL = phosphoglyceraldehyde, PPP = pentose phosphate pathway, Pro = proline, PYCR1-3 = pyrroline-5-carboxylate reductase 1-3.

The metabolism branch known as the pentose phosphate pathway (PPP) plays a role in the development of MM, being connected to the differential expression of serum metabolites of glucose-6-phosphate (G6P) and dehydroepiandrosterone sulfate. Enhanced G6PD expression leads to increased production of the antioxidant NADPH within the PPP, consequently reducing the generation of ROS, thereby promoting the proliferation of MM cells^[45]^ (Fig. 4). Targeting G6PD to exploit cellular redox may serve as an innovative approach to managing MM. Metabolomic analysis has shown that BCR-ABL-ERK signaling drives the PPP and RNA biosynthesis in myeloma cells and that imatinib inhibits glycolysis and PPP uptake of glucose via acetyl coenzyme A and NADPH resulting in PPP damage and RNA nucleotide accumulation and negative regulation of mRNA.^[46]^ Furthermore, Bone destruction is also a feature of myeloma, and osteoclasts are dependent on glucose metabolism on glycolysis and oxidative phosphorylation (OXPHOS), playing a key role in lactic acid production^[47]^ (Fig. 4). Therefore, glycolytic pathways, especially the PPP, can provide a good direction for the diagnosis and treatment of the MM.

Proteasome inhibitors are the main therapeutic drugs for MM, such as bortezomib. Studies have indicated that elevated glycolytic activity or altered glucose metabolism is linked to the development of resistance to bortezomib in MM.^[48,49]^ Despite numerous efforts to block the elevated glycolysis seen in MM, the presence of conserved glycolytic pathways in both normal and MM cells, along with the existence of alternative metabolic routes, has hindered the success of clinical interventions. This underscores the pressing demand for effective treatments^[50]^ (Fig. 4).

### 4.2. Amino acid metabolism

MM cells are strictly glutamine dependent and exhibit glutamine addiction, with enhanced glutamate metabolism in BM, with upregulated glutamate levels and downregulated glutamine levels, providing ammonium (NH4+) for ammonia utilization, resulting in elevated levels of urea and creatinine from the urea cycle, arginine and proline metabolism. This is also accompanied by NH4 + accumulation, which is released into the circulation, thereby increasing the chance of hyperammonemia, and inhibition of glutamine uptake may be a therapeutic strategy for MM (Fig. 4). Interestingly, a recent investigation revealed that alterations in the metabolism of myeloma cells trigger reciprocal changes in bone cell activity, and myeloma cell consumption of glutamine leads to an increase in glutamine synthetase in bone marrow stromal cells and inhibits their osteoblast differentiation.^[51]^ Furthermore, the labeling of glutamine with 13C revealed a noticeable rise in glutamine reentry into the TCA cycle in plasma cells of myeloma patients.^[52,53]^ Meanwhile threonine was identified as a priority plasma biomarker for risk prediction.^[51]^ Other studies using ultra performance liquid chromatography coupled with high resolution orbital trap mass spectrometry Q Exactive™ (UPLC-MS) found that aberrant glycine, serine and threonine metabolism, glycerophospholipid metabolism, as well as NF-κB and PI3K/Akt/mTOR signaling pathways were activated in MM cells, inducing the secretion of large amounts of IL-6 and growth factors by BM stromal cells, promoting the growth, proliferation and survival of myeloma cells^[54]^ (Fig. 4). These novel insights into the metabolic adaptability of myeloma cells are anticipated to introduce fresh avenues for therapeutic strategies.

Glycine dehydrogenase has been found to promote GSH production, and knockdown of glycine dehydrogenase induced DNA damage, thereby inhibiting MM cell proliferation.^[55]^ Previous publications have found that exogenous glycine only contributes to purine synthesis in melanoma.^[56]^ In contrast, it has been observed in a recent investigation that MM cells uptake exogenous glycine and further metabolize it into GSH, purines, and serines.^[55]^ In summary, the increased presence of glycine within the BM contributes to the progression of MM by modulating the GSH equilibrium within MM cells. The therapy involving the restriction of glycine utilization through betaine has displayed promising treatment outcomes for MM, either individually or when combined with bortezomib.^[50,55]^ Furthermore, the upregulation of pyrroline-5-carboxylate reductase 1-3 (PYCR1-3) and pyrroline-5-carboxylate synthase is induced by MYC, leading to the promotion of glutamine to proline biosynthesis.^[44,57]^ Interfering with PYCR1 leads to a decrease in myeloma cell proliferation and survival by blocking the pathway of proline-rich akt substrate 40 kDa-mediated protein synthesis. PYCR1 inhibition reduces myeloma cell proliferation and survival by triggering unfolded protein response pathway in vitro and in vivo to increase the efficacy of bortezomib^[44]^ (Fig. 4). Thus PYCR1 overexpression is related to the progression of several cancers, and reducing PYCR1 level can reduce tumorigenicity, providing a new idea for the precise treatment of diseases.

### 4.3. Lipid metabolism

Myeloma cells exhibit a notable increase in heat shock protein 60 levels, it inhibits adenosine 5′-monophosphate (AMP)-activated protein kinase (AMPK), while AMPK inhibits the key enzyme 6-phosphofructo-2-kinase/fructose-2,6-bisphosphatase 3 (PFKFB3), which affects FA metabolism coenzyme A by inhibiting malondiyl.^[58]^ Interestingly, experimental studies have found that abnormal glycerophospholipid metabolism induces the secretion of large amounts of IL-6 and growth factors by BM stromal cells, promoting the growth, proliferation and survival of myeloma cells^[54]^ (Fig. 4). In conclusion, abnormalities in lipid metabolism lead to the development of diseases, and the study of lipid metabolism can provide a common direction for the diagnosis and treatment of MM.

## 5. Metabolomics and leukemia

Leukemia is a type of cancer characterized by immune cell depression and destruction of normal BM function. It encompasses various types such as acute myeloid leukemia (AML), acute lymphoblastic leukemia (ALL), chronic myeloid leukemia (CML), and CLL, each presenting unique challenges and characteristics.^[59]^ Research findings have indicated that the altered metabolic processes play a crucial role in the initiation and advancement of leukemia, making the metabolic activities of leukemia cells a prime target for potential treatments. A potential avenue for therapeutic intervention lies in targeting the altered metabolism believed to accompany the development of drug resistance in leukemia cells.

### 5.1. Metabolomics in acute myeloid leukemia

**Figure 5.**
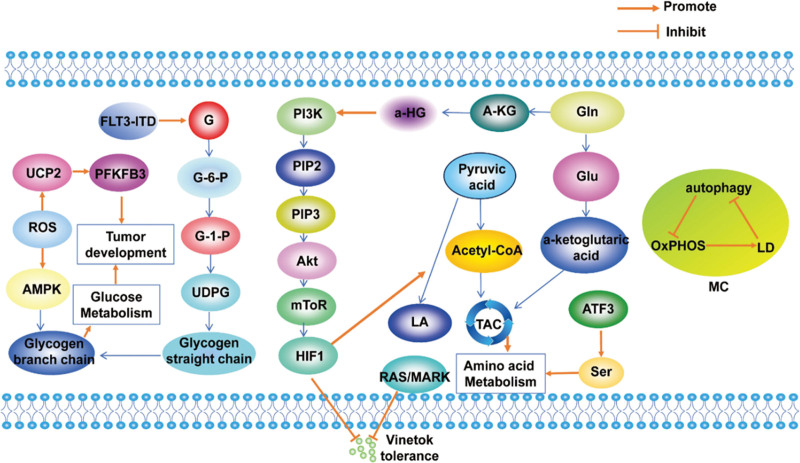
Glucose, amino acid and lipid metabolism in AML. Synthesis of glycogen activates AMPK, leading to growth inhibition of leukemic cells, and ROS promotes UCP2 protein expression and AMPK phosphorylation, upregulating the expression of the key regulatory glycolytic enzyme PFKFB3. Downregulation of PFKFB3 strongly inhibits leukemia cells growth in vitro and in vivo. PI3K/Akt/mTOR promotes the glycolytic pathway, leading to lactate production. as glutamine is a source of α-KG, which can be converted to 2-HG by IDH mutants, cycling back and forth to promote leukemogenesis. Autophagy leading to a decrease, in OXPHOS, OXPHOS support the proliferation and growth of leukemia through lipid degradation. a-KG = A-ketoglutaric acid, ATF3 = activating transcription factor 3, G = glucose, Gln = glutamine, Glu = glutamic acid, LA = lactic acid, TAC = tricarboxylic acid cycle, UCP2 = uncoupling protein 2, UDPG = uridine diphosphate glucose.

AML presents as a malignancy affecting the hematopoietic system, characterized by the accumulation of primitive myeloid cells in the bone marrow and peripheral blood. Resistance to common induction therapies, like doxorubicin and arachidonic acid (Ara-C), is inevitable in 35% to 45% of newly diagnosed AML patients.^[60]^ Dysregulated cellular metabolism has become the focus of research and therapeutic interventions in AML.^[61]^ At the intracellular level, AML has been reported to exhibit dysregulation of amino acid, nucleotide, lipid and bioenergetic metabolism.^[62]^ Wang et al And Musharraf et al by comparing the metabolic profiles between individuals with AML and those without the disease reveals significant deviations in pathways such as the TCA cycle, glycolysis, and FA metabolism^[63,64]^ (Fig. 5). The metabolics highlights the potential clinical application value in the treatment of AML.

#### 5.1.1. Glucose metabolism

Leukemia cells are characterized by an increase in glucose uptake and enhanced aerobic glycolysis, which have been well-documented. The dysregulation of glucose metabolism is closely linked to resistance to treatment and the overall clinical prognosis. Bhanot et al^[65]^ validated that the glycogen precursor UDP-D-glucose exhibited an increase in AML cells, indicating a critical reliance on heightened glucose metabolism for cell proliferation. Data from cancer genomics has indicated a correlation between heightened levels of glycogen synthase 1 and 2 (GYS1/2) or glycogen branching enzyme 1 with unfavorable outcomes in AML patients. When focusing on glycogen synthase 1, a reduction in glycolytic flux was observed alongside an elevation in the activation of glycogen-responsive AMPK, ultimately resulting in significant growth suppression in leukemia cells. Furthermore, the elevation of UCP2 protein expression, AMPK phosphorylation and upregulation of PFKFB3(a key regulatory glycolytic enzyme) induced by ROS. The growth of leukemia cells in vitro and in vivo is significantly suppressed with the decrease in PFKFB3 activity.^[66]^ Therefore, studying the inhibitors of this protein may provide easily addressed therapeutic targets in AML. Internal tandem repeats (ITD) within the proximal membrane structural domain of FLT3 represent poor prognostic indicators in AML, and a global non-targeted metabolomic approach has been applied to analyze metabolite differences between sorafenib-sensitive and drug-resistant leukemia cells with FLT3-ITD mutations and found that glycolytic activity was enhanced, accompanied by a disturbed TCA cycle and reduced PPP flux rate, as well as an increased antioxidant capacity of GSH (Fig. 5).

In clinical settings, the FDA has recently granted approval for the utilization of Venetoclax, a specific inhibitor of B-cell lymphoma 2, when combined with low-dose cytarabine or demethylating agents in the elderly AML population or those unsuitable for chemotherapy (Fig. 5). Nevertheless, AML patients often face challenges due to the development of resistance towards venetoclax. Non-targeted metabolomics data showed that drug resistance resulted in significant alterations in the PI3K/Akt pathway, Warburg effect, glycolysis, TCA cycle, and REDOX metabolism.^[67]^ Numerous research studies have highlighted substantial alterations in the metabolic characteristics of drug-resistant cells due to a variety of external elements influencing the metabolic transformations. Factors such as tyrosine kinase receptors, G protein-coupled receptors, and different growth factors have been linked to the activity of the PI3K/Akt/mTOR pathway, Upon activation of the PI3K signaling cascade, the phosphorylation of phosphatidylinositol 4,5-bisphosphate gives rise to the second messenger phosphatidylinositol 3,4,5-trisphosphate (PIP3),^[67]^ this, in turn, facilitates the activation of the Ser/Thr kinase Akt (protein kinase B), a critical downstream target of PI3K. On the contrary, Akt is localized in the cytoplasm in inactive cells, necessitating PI3K activation to transport Akt to the membrane (where PIP3 serves as a docking site).^[68]^ Subsequently, mTOR is activated downstream by Akt, which is in turn activated by PIP3.^[69]^ The PI3K/Akt/mTOR pathway is known to regulate cellular metabolism through a different mechanism. Activation of mTOR results in the induction of hypoxia-inducible factor-1, it stimulation enhances the glycolytic pathway, resulting in the production of lactate. It has been noted that the resistance to vinetoclax by enhancing MCL-1 through the RAS/MAPK pathway, thereby inducing metabolic routes that support OXPHOS like FA and amino acid metabolism, plays a role in providing resistance to vinetoclax in AML^[70]^ (Fig. 5). In the MV4-11 ABT199-resistant strain (ABT199-r), the reduction of phosphorylated forms of phosphatidylinositol was achieved by inhibiting the PI3K/Akt/mTOR signaling pathway using PIK-402 inhibitors. Following this, the levels of GLUT-3 and metabolic pathways associated with glycolysis saw a decline. Thus, vinetoclax resistance is associated with activation of the PI3K/Akt pathway, and treatment of ABT199-R with PIK-402 transforms metabolism from glycolysis to OXPHOS. In addition, altered electroglycan metabolism is strongly associated with treatment resistance and clinical outcomes.^[67]^ It has been shown that enhanced glycolysis contributes to reduced sensitivity to the anti-leukemic drug Ara-C, while inhibition of glycolysis inhibits AML cell proliferation and enhances the cytotoxicity of Ara-C^[71]^ (Fig. 5). Therefore, metabolomics is emerging as a powerful approach to study cancer metabolism, and systemic metabolic markers, such as altered glucose metabolic profile, are associated with the prognosis of AML.

#### 5.1.2. Amino acid metabolism

Glutamine is the primary metabolite essential for cell growth in AML and ALL, with its abundance in the blood and muscle as the most prevalent circulating amino acid, and it undergoes metabolic processes within the mitochondria, following the enzymatic pathway of glutamine breakdown. In fact, following its entry into the cell through specific transporter proteins like ASCT2 and SN2 from the sodium-dependent neutral amino acid transporter type 2 and solute carrier family 38 member 5, respectively, the process of converting it to glutamate is carried out by glutaminase, followed by the transformation of glutamate to α-ketoglutarate via glutamate dehydrogenase or transaminases, which in turn contributes to the TCA cycle through a mechanism known as back-completion, this process enables the maintenance of mitochondrial stability and operation in conditions where pyruvate availability is limited due to aerobic glycolysis.^[72]^ Glutamine plays a vital role in the production of GSH, thus playing a crucial role in regulating redox homeostasis in acute myeloid leukemia, with a specific focus on its contribution to metabolic reprogramming, this metabolite is utilized by leukemia cells as a carbon source to support energy production through the TCA cycle and maintenance of redox balance, which plays a crucial role in cell viability, and the role of glutamine as a precursor of α-KG is crucial, this substance has the potential to be transformed into 2-HG by IDH mutants, This bidirectional flow facilitates the progression of leukemia.^[68]^ The above studies on amino acid metabolism provide a solid theoretical basis for promoting the mechanisms of AML development and finding new therapeutic targets (Fig. 5).

The breakdown of glutamine plays a vital role in supporting the growth and survival of AML cells.^[68]^ Zavorka Thomas et al^[73]^ identified a crucial metabolic route for the proliferation and viability of AML cells. The inhibition of tyrosine kinase by gilteritinib was observed to result in a decrease in glutamine metabolism through the TCA cycle, alongside a reduction in cellular levels of the oncogenic metabolite 2-hydroxyglutarate. Treatment with Gilteritinib resulted in a decline in cellular ATP generation and glutathione (GSH) synthesis, along with an elevation in reactive oxygen species, culminating in the induction of cellular senescence. Activation of glutamine metabolism provides protection to AML cells during times of extreme stress following treatment, indicating the potential to disrupt glutamine metabolism in order to eradicate persistent AML cells.^[74]^ The coordination of serine synthesis and catabolism, as well as purine and pyrimidine synthesis by the transcription factor activating transcription factor 3, is pivotal in supporting AML cell cycling, survival, and differentiation blockade. Activating transcription factor 3 serves as an unidentified controller of serine and nucleotide metabolism, paving the way for potential therapeutic targets in the context of AML^[75]^ (Fig. 5). These results offer a conceptual foundation for crafting targeted combined treatment approaches aimed at a subgroup of relapsed/refractory AML.

#### 5.1.3. Lipid metabolism

Taking into account the existing clinical diagnostic techniques, Metabolic pathways based on differential metabolite and lipid profiles offer insights into various types of leukemia, enhancing our understanding of leukemia’s physiological characteristics.^[76]^ It is recognized that autophagy serves as a vital survival mechanism enabling cells to adapt to challenging growth environments, playing a role in tumorigenesis. Studies have shown that blocking autophagy can lead to an increase in lipid droplets formation as a result of reduced FA β-oxidation, resulting in a reduction of oxidative phosphorylation specifically in AML cells. The analysis of the mechanism revealed that oxidative phosphorylation supports the proliferation and expansion of leukemia by breaking down lipids, this process was influenced by autophagy regulation^[77]^ (Fig. 5). In conclusion, focused on the regulation of lipid metabolism, with a particular emphasis on autophagy, represents a promising avenue for further exploration in the field of disease treatment possibilities.

### 5.2. Acute lymphoblastic leukemia

ALL is the most frequent childhood cancer, accounting for 80% of childhood leukemias.^[78]^ Glucocorticoids have shown great efficacy in treatment. However, in B-cell precursor ALL, around 20% of patients experience relapse and succumb to the disease. Survivors often endure long-term negative effects from therapy, with glutamine synthesis affecting autophagy and possibly GC-induced cell death.^[79]^ As a result, it is crucial to have a thorough grasp of the disease’s metabolic profile and actively seek out novel metabolic targets.

#### 5.2.1. Glucose metabolism

**Figure 6.**
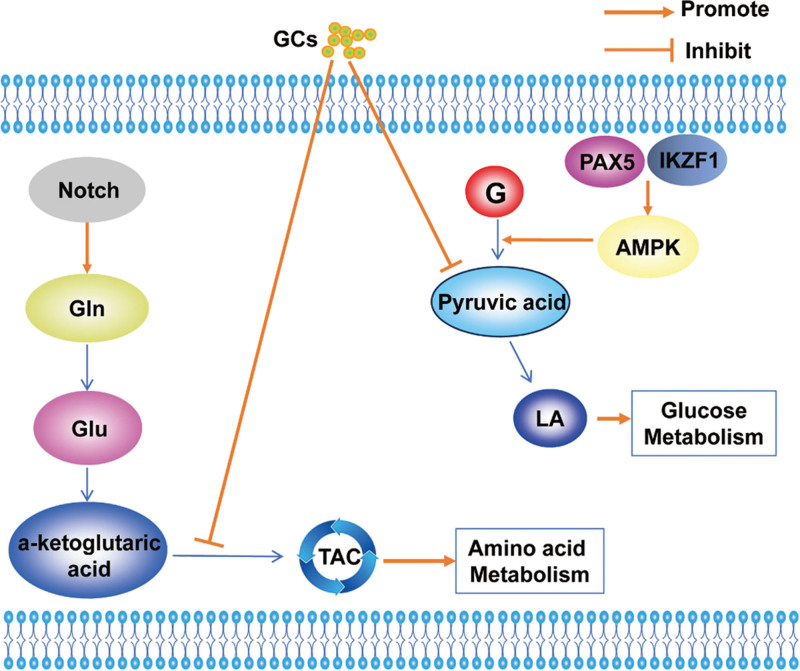
Glucose and amino acid metabolism in ALL. GCs not only inhibit glycolysis, but also inhibit the entry of glucose and glutamine into the TCA cycle. Upregulation of Notch1 accelerates the glutamine catabolism, activates cell growth signaling and blocks glutamine anabolism. ALL = acute lymphoblastic leukemia, GCs = glucocorticoids, IKZF1 = IKAROS family zinc finger 1, Notch1 = notch receptor 1, PAX5 = paired box 5, TCA = tricarboxylic acid.

Key transcription factors in B lymphocytes, such as paired box 5 and IKAROS family zinc finger 1, play a crucial role in the early development of B cells. Through advanced techniques like ChIP-seq and RNA sequencing, a new program specific to B lymphocytes has been uncovered. This program works to suppress the transcription of genes related to glucose and energy supply, inducing a constant state of energy scarcity, ultimately activating the AMPK energy stress sensor. And it was found that heterozygous deletion of Pax5 increased glucose uptake and ATP levels > 25-fold in B-cell precursor ALL mouse model^[80]^ (Fig. 6). Therefore, strengthening the study of the above pathways and the study of related transcription factors could bring hope for the treatment of ALL patients and provide a major step toward precision therapy.

Clinically, Glucocorticoids have been shown to be effective agents for the treatment of childhood ALL. Inhibiting not only glycolysis but also the TCA cycle’s uptake of glucose and glutamine has been observed. It has also been revealed by a combination of transcriptomic and metabolomic analyses that cells resistant to erythromycin are more glucose-dependent and less glutamine- and FA-dependent, and that a better understanding of the metabolism of resistance could provide guidance for the discovery of biomarkers for therapeutic selection and the development of new therapeutic strategies that could potentially give new mandates to old drugs^[81]^ (Fig. 6).

#### 5.2.2. Amino acid metabolism

It has been shown that upregulation of notch receptor 1 (Notch1) accelerates the glutamine catabolism, activates cell growth signaling and blocks glutamine anabolism, ultimately leading to Notch1-driven glutamine addiction in T-ALL^[82]^ (Fig. 6).

### 5.3. Metabolomics in chronic myeloid leukemia

Chronic granulocytic leukemia (CML) is a malignant clonal disease of hematopoietic stem cells that results in an decline in myeloid and erythroid cells and platelets in the peripheral blood. Imatinib is a BCR-ABL1-specific tyrosine kinase inhibitor (TKI) and is considered the preferred treatment for the treatment of CML. Despite promising results with standard doses of imatinib as the treatment strategy of choice for patients with chronic CML, approximately 25% of patients eventually develop resistance to imatinib. To overcome this resistance, second-generation TKI (i.e. dasatinib and nilotinib) have been developed, but their resistance remains a clinical problem. Metabolomics approaches may help to identify novel mechanisms of CML pathogenesis and progression. Metabolomic approaches may help to identify new mechanisms of CML pathogenesis and progression.

#### 5.3.1. Glucose metabolism

**Figure 7.**
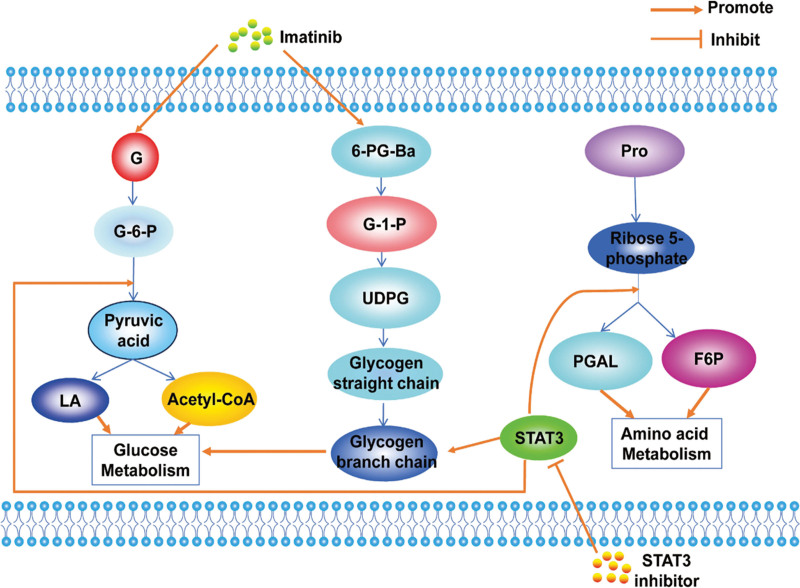
Glucose, amino acid and lipid metabolism in CML. Long-term imatinib-resistant cells increase glycolysis and PPP. Inhibition of STAT3 by STAT3 inhibitor sensitized CML cells to TKI treatment. Dysregulation of SATA3 mitochondrial metabolism led to increased dependence on glycolysis in tki persistence CML cells, while targeting pyruvate kinase M2 (a rate-limiting glycolytic enzyme) specifically eradicated tki persistence CML cells. CML = chronic granulocytic leukemia, PGAL = 3-phosphoglyceraldehyde, PPP = pentose phosphate pathway, STAT3 = signal transducer and activator of transcription 3, TKI = tyrosine kinase inhibitor.

The crucial involvement of Signal transducer and activator of transcription 3 (STAT3) in the initiation and progression of CML has been well documented. Sweta B. Patel et al Explored the impact of STAT3 on inducing metabolic alterations in TKI persistent CML cells. Following the knockout of STAT3, a significant inhibition in the drug persistence of CML cells was observed, leading to an increased sensitivity of persistent CML cells to TKI therapy upon inhibition of STAT3 with small molecule inhibitors. Finally, it was discovered that the disruption of mitochondrial metabolic regulation by STAT3 leads to an increased reliance on glycolysis in TKI persistent CML cells, and the targeted inhibition of pyruvate kinase M2, a key glycolysis-regulating enzyme, effectively eliminates these cells. Investigating the impact of STAT3 on metabolic changes offers crucial insights into pinpointing effective treatment targets to eradicate TKI-persistent LSC^[83]^ (Fig. 7). Examining how glucose pathways are diversified into various metabolic routes, it was observed that resistant cells to TKI treatment rely more on glycolysis as they develop resistance. Moreover, it was discovered that the utilization of glucose becomes interconnected with alternative pathways distinct from glycolysis, such as PPP, this indicates that CML cells have a preference for converting glucose through glycolysis into lactic acid, while those resistant to long-term imatinib treatment exhibit elevated levels of glycolysis and PPP^[84]^ (Fig. 7).

#### 5.3.2. Amino acid metabolism

The results show that single-carbon metabolism and GSH metabolism inhibition are highly effective in targeting CML cells, suggesting that methotrexate, Pemetrexed, and RSL-3 could be explored as potential strategies for managing TKI resistance in the future.^[84]^ Increased research on amino acid metabolism could reveal mechanisms of drug resistance and thus suggest more precise therapeutic measures (Fig. 7).

#### 5.3.3. Lipid metabolism

Utilizing liquid chromatography, mass spectrometry, and bioinformatics analysis of plasma metabolites in patients with advanced CML revealed a reprogramming of lipid metabolism in these individuals. Specifically, drug-resistant patients exhibited elevated levels of ceramide and reduced levels of sphingomyelin^[85]^(Fig. 7). This opens up a new avenue for considering the treatment of advanced CML patients.

### 5.4. Metabolomics in chronic lymphocytic leukemia

**Figure 8.**
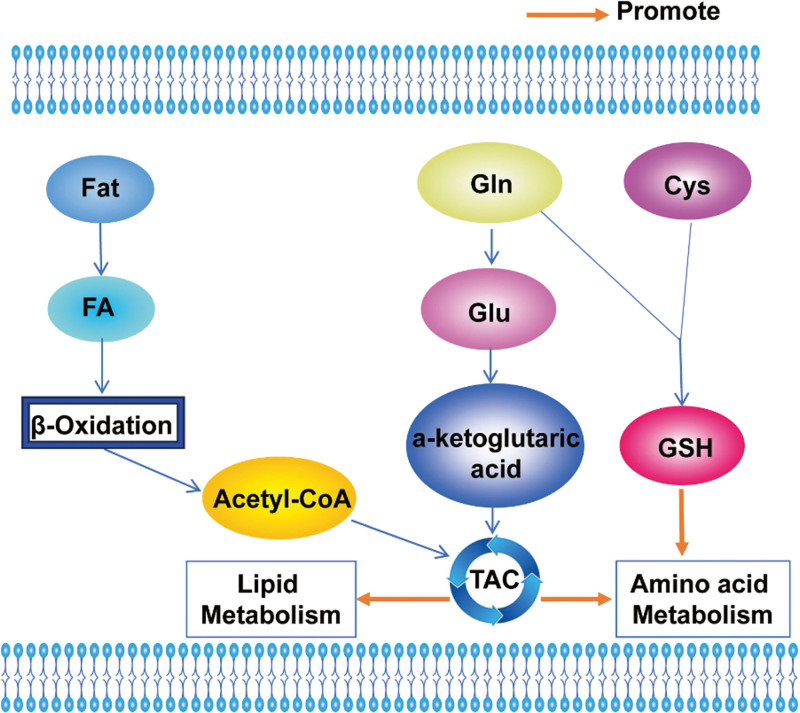
Glucose, amino acid and lipid metabolism in CML. CLL patients have increased glutamine consumption and increased β-oxidation of FA. CML = chronic granulocytic leukemia, CLL = chronic lymphocytic leukemia, Gln = glutamine, Glu = glutamic acid, FA = fatty acid.

B-cell chronic lymphocytic leukemia (B-CLL) is the most common type of leukemia in adults and remains largely incurable despite the development of new treatment strategies. Research conducted by Vangapandu et al^[86]^ examined the mitochondrial OXPHOS and glycolysis in primary CLL cells co-cultured with various stromal cell lines. It was observed that the OXPHOS levels were notably elevated in CLL cells co-cultured with BM-derived NK stromal cells compared to CLL cells cultured in isolation, the extracellular acidification rate, an evaluation of glycolytic activity, in a study on CLL cells cocultured with stromal cells, diverse changes were observed. An analysis of metabolomics profiles and the quantitation of ribonucleotide pools indicated the potential induction of CLL cellular bioenergy in this coculture setting. Additionally, a proteomic analysis by Rupert L Mayer et al on primary human B-CLL cells and B cells from healthy donors revealed significant alterations in amino acid and lipid metabolism in B-CLL cells. including upregulation of alanine and downregulation of glutamine, Glutamic acid, serotonin Furthermore, there was also an increase in the levels of various phosphatidylcholines and sphingomyelins observed. These findings collectively suggest a heightened consumption of glutamine and enhanced β-oxidation of FA in individuals diagnosed with CLL.^[87]^ The exploration of metabolomics in CLL remains limited and warrants additional investigation for potential new therapeutic targets (Fig. 8).

## 6. Research criteria and methods

When designing a metabolomics study, it is crucial to reduce the impact of specific factors, such as age, gender, fasting status, dietary habits, and the timing of sample collection. To prevent the formation of compounds resulting from multiple freeze-thaw cycles, it is essential to store samples in separate aliquots immediately after collection.^[88]^ Factors used to process samples (e.g., sample preparation, pH buffering, and extraction) should be carefully controlled and standardized to ensure consistent results.^[89]^ Comparison of serum metabolomics data from high-risk patients with serum metabolomics data from patients cured by standard chemotherapy may provide useful information about patient prognosis and mechanisms of treatment failure.^[90]^ After generating metabolomics data, it is important to ensure their reproducibility.^[88]^ The processing of data and subsequent statistical analysis are carried out utilizing data analytics and bioinformatics. One popular method for analyzing multivariate data statistically involves the distinction between unsupervised learning and supervised learning. A widely used method in unsupervised learning is the application of principal component analysis (PCA). Another major methodology is utilizing supervised learning techniques, like artificial neural networks (ANN) and partial least squares discriminant analysis (PLS-DA), to further explore biomarkers within the dataset.^[91]^ Supervised models play a crucial role in driving the discovery of biomarkers, linking them to clinical outcomes, histopathology scores, and a range of other histologic data.

The levels of metabolites in patients with different hematologic malignancies are different from those in healthy individuals, and rise or fall are closely related to disease. Below are the changes in metabolites in the blood and urine of patients with clinical hematologic malignant neoplasms tested compared to healthy individuals (Table 1).

**Table 1:** Metabolites change in the patient.

Tumor	Metabolic type	Metabolite changes in patients	Reference
Lymphoma	Glucose metabolism	Rise: Fumarate; uridin; S-adenosylmethioninamine Rise: lactate; glucuronic acid Descend: succinic acid Rise: lactate Descend: glucose	^[16]^ ^[17]^ ^[36]^
	Amino acid metabolism	Rise: glutathione Rise: tryptophan metabolites Descend: alanine; isopropylmalic acid; L-Valine; L-serine Rise: glycine Descend: hypoxanthine Descend: Gln; glucose; serine Descend: Gln Rise: pyruvate	^[16]^ ^[17]^ ^[18]^ ^[30]^ ^[31]^ ^[42]^
	Lipid metabolism	Rise: Choline; D-Galactose Rise: FA Rise: Choline Rise: glycerol; FA	^[16]^ ^[21,32]^ ^[23,32]^ ^[32,38]^
Myeloma	Glucose metabolism	Rise: Fumarate; uridin; S-adenosylmethioninamine	^[16]^
	Amino acid metabolism	Rise: glutathione Rise: Uric acid/ Linoleic acid/palmitic acid; Rise: G6P; Rise: Glu; urea; creatinine Descend: Gln; threonine Descend: Gln Rise: IL-6 and growth factors Descend: glycine, serine and threonine/	^[16]^ ^[18]^ ^[45]^ ^[51]^ ^[52,53]^ ^[54]^
	Lipid metabolism	Rise: Phosphatidylcholine	^[16]^
Acute myeloid leukemia	Glucose metabolism	Rise: UDP-D-glucose; glycogen synthase 1 and 2 (GYS1/2) or glycogen branching enzyme 1 (GBE1)	^[65]^
	Amino acid metabolism	Rise: hypoxanthine Rise: α-KG Descend: Gln	^[18]^ ^[68]^
Acute lymphoblastic leukemia	Amino acid metabolism	Rise: hypoxanthine Rise: Gln	^[19]^ ^[82]^
Chronic lymphocytic leukemia	Amino acid metabolism	Rise: Hypoxanthine; Uric acid; Rise: alanine; glutamine; glutamic acid; serotonin; phosphatidylcholines: sphingomyelins	^[18]^ ^[87]^

## 7. Summary and outlook

The increasing annual rate of hematological tumors in recent years has had a significant impact on the health and quality of life of individuals. Metabolomics involves the in-depth examination of metabolites and various small molecules found in tissues, blood, and biological samples. Being a recent addition to the field of systems biology, it is expected to find diverse applications in the investigation of hematological malignancies. By exploring the potential correlation between molecular defects and metabolite alterations, establishing a macroscopic system model from genetic material, functional material to end products, and finally building a comprehensive understanding of the disease at the level of systems biology, screening metabolites that are important to the disease, discovering molecular markers with early diagnosis, efficacy evaluation and prognosis estimation values, providing early diagnosis, efficacy evaluation, accurate staging and individualized targeting for the disease. The development of this field faces inherently different challenges, including the identification process. Despite the use of common elements such as C, H, O, N, S, P, and potentially heteroatoms, these small molecules do not possess a uniform basic structure. The assumption often made is that MS-based metabolomic analysis will generate a considerable number of small molecules labeled as “identified,” which can be associated with networks and pathways. However, the metabolomic identification process poses a significant challenge, making high confidence analyte assignment or identification difficult. Computerized metabolite databases may offer some assistance and occasionally validation, yet they may not be applicable to every metabolomics investigation. Validation of retention times and MS/MS fragmentation data using reference standards is typically essential for the reliable identification of metabolites. Understanding the complexities associated with LC-Ms-based metabolomics and the level of certainty involved in characterizing small molecules during comprehensive metabolomic analyses is crucial for leveraging metabolomic information to gain insight into human health and disease mechanisms. An extensive and methodical exploration of metabolomics, encompassing glucose metabolism, amino acid metabolism, and lipid metabolism, lays a foundation for the timely detection, assessment of effectiveness, precise classification, and individualized targeted therapy of hematological tumors.

## Author contributions

**Conceptualization:** Yanying Chen.

**Data curation:** Yanying Chen.

**Formal analysis:** Yongqing Zhai.

**Funding acquisition:** Lijuan Wang.

**Investigation:** Mengyu Xu.

**Methodology:** Mengyu Xu.

**Project administration:** Yongqing Zhai, Jiawei Yin, Lijuan Wang.

**Resources:** Yanying Chen.

**Software:** Junhong Li, Ning Zhang.

**Supervision:** Jiawei Yin.

**Validation:** Yongqing Zhai, Ning Zhang, Lijuan Wang.

**Visualization:** Ning Zhang, Jiawei Yin, Lijuan Wang.

**Writing – original draft:** Xinglan Li.

**Writing – review & editing:** Xinglan Li.
